# Sleep-Related Offline Improvements in Gross Motor Task Performance Occur Under Free Recall Requirements

**DOI:** 10.3389/fnhum.2016.00134

**Published:** 2016-03-29

**Authors:** Andreas Malangré, Klaus Blischke

**Affiliations:** Sport Science, Training Science, Saarland UniversitySaarbruecken, Saarland, Germany

**Keywords:** sleep, enhancement consolidation, gross motor task, sequence learning, free recall

## Abstract

Nocturnal sleep effects on memory consolidation following gross motor sequence learning were examined using a complex arm movement task. This task required participants to produce non-regular spatial patterns in the horizontal plane by successively fitting a small peg into different target-holes on an electronic pegboard. The respective reaching movements typically differed in amplitude and direction. Targets were visualized prior to each transport movement on a computer screen. With this task we tested 18 subjects (22.6 ± 1.9 years; 8 female) using a between-subjects design. Participants initially learned a 10-element arm movement sequence either in the morning or in the evening. Performance was retested under free recall requirements 15 min post training, as well as 12 and 24 h later. Thus, each group was provided with one sleep-filled and one wake retention interval. Dependent variables were error rate (number of Erroneous Sequences, ES) and average sequence execution time (correct sequences only). Performance improved during acquisition. Error rate remained stable across retention. Sequence execution time (inverse to execution speed) significantly decreased again during the sleep-filled retention intervals, but remained stable during the respective wake intervals. These results corroborate recent findings on sleep-related enhancement consolidation in ecological valid, complex gross motor tasks. At the same time, they suggest this effect to be truly memory-based and independent from repeated access to extrinsic sequence information during retests.

## Introduction

There is ample evidence by now that sleep (but not wake) after initial training of motor skills can produce significant improvements in performance at later retesting without any further physical practice (e.g., Fischer et al., 2005; Walker, 2005; Doyon et al., 2009). This phenomenon usually is referred to as “sleep-related offline learning”, and has been associated with an “active system consolidation” process (Born and Wilhelm, 2012). Here, it is assumed that newly encoded skill representations are being actively reprocessed during slow-wave sleep, resulting in strengthening synaptic connections in the neocortex and in a qualitative reorganization of the respective memory representations. These processes are understood as a prerequisite for the sudden improvements in overt performance frequently observed. However, suchlike processes and effects appear to be closely related to certain motor task characteristics as well as to specific learning procedures. That is, in general sleep-related offline learning seems to require some involvement of *declarative* memory processes. This is often associated with routines of *explicit* learning and awareness (Robertson et al., 2004). Enhancement of motor sequence memory supposedly pertains to an abstract spatial map of the sequence that represents the series of movements to perform during recall. This representation is supported by a distinct hippocampo-cortical neural network (Albouy et al., 2015), and is supposedly associated with declarative knowledge concerning the action’s goal as well as the type of sequence elements and their temporal order. Moreover, performance improvements in motor adaptation tasks (i.e., precise sub-maximal force production; visuo-motor adaptation) have been found to be fairly small and rather *time*- instead of sleep-dependent (Blischke et al., 2008; Doyon et al., 2009; but see Huber et al., 2004). Thus, sleep-related EC should be most pronounced in *sequentially* structured motor tasks.

In most of the studies addressing sleep-related motor offline learning sequential-finger-tapping or thumb-to-finger movements were involved. Only a couple of years ago the question has been raised if the respective findings also apply to ecologically valid gross motor skills (Blischke et al., 2008). And it was only recently that these findings have successfully been extended to gross motor tasks involving multi-joint limb movements (Genzel et al., 2012; Kempler and Richmond, 2012; Morita et al., 2012; Al-Sharman and Siengsukon, 2013; Malangré et al., 2014). Moreover it has been shown that the degree of sleep-related motor enhancement consolidation in the elderly is modulated by the kinematic demands of the task. In one recent study, sleep-related performance improvements were observed in older age groups only when a classic sequence learning task requiring individuated finger movements was replaced by an adapted version of the same task. In this adapted version reaching movements were performed with the whole hand (Gudberg et al., 2015). This dissociation of specific mechanisms of sleep underpinning motor sequence consolidation in older adults is certainly of theoretical importance. And it emphasizes the potential of incorporating whole limb movements in research activities concerning the relation of sleep and motor memory consolidation.

Although criterion tasks incorporated in all these studies reporting gross motor sleep-related offline learning were of considerable variety and involved movements of the upper as well as of the lower extremities, again they were all *sequentially* structured. However, when motor *adaptation* was the prominent task requirement, sleep did not enhance, but only stabilize performance (Hoedlmoser et al., 2015). Thus, the above mentioned dissociation of motor sequence learning and motor adaptation with respect to sleep-related memory consolidation processes seems to hold also for gross motor skills. However, there are still some aspects of practical and theoretical importance waiting for closer scrutiny. One such aspect is the question as to whether sleep-related offline learning will also come into effect at retention even under *free recall* conditions at an early learning stage. This question is of particular importance in the applied field of movement studies (i.e., vocational training, sports, occupational therapy, and motor rehabilitation). Here trainees, athletes and patients initially are supplied with stimulus information and feedback while acquiring new motor skills at initial training sessions. But soon after initial training they are usually required to recall and execute those skills under “real-life” conditions in the absence of any augmented information.

Here as a first step we present an experiment set up to scrutinize if sleep-related offline learning was to be found at all in a gross motor task under *free recall* conditions with no extrinsic criterion information available. The criterion movement employed was a sequential motor task with high demands on precision and manual dexterity. This task incorporated a series of 10 unrestrained multi-joint reaching movements involving the whole non-dominant arm. Such a task bears good resemblance to a wide variety of sport skills and activities of daily living. Following a fixed spatial pattern, participants had to execute this movement sequence as rapidly as possible with as few errors as possible.

It was hypothesized that after initial learning *sleep*, but not wake, significantly facilitates performance (namely: sequence execution speed) at retention under free recall conditions when compared to post-training performance (i.e., free-recall performance assessed shortly after acquisition).

## Materials and Methods

### Participants and Groups

Two groups of participants (*N* = 12 each) voluntarily participated in this experiment, which was conducted at the Saarland University (Department of Sport Science) in accordance with the ethical standards of the 1975 Declaration of Helsinki, and was approved by the Ethic Committee of the Faculty 5 Empirical Social Sciences of Saarland University. Subjects took part in the experiment in accord with the department’s course regulations and gave their written informed consent before participation. Participation was accounted for as partial fulfilment of course requirements. For organizational reasons both groups were recruited from different courses, and were examined at different times about 6 months apart by different experimenters.

Six subjects did not complete the experiment, because they were unable to recall the criterion task under free recall conditions. These subjects were excluded from further analysis. Only the remaining 18 participants entered the final analyses reported in the following sections. As a consequence the first group (in the following labeled the Morning-Evening-Morning (MEM) group according to the experimental design; cf. “Design and Procedure” Section) comprised only 8 participants (22.1 ± 2.4 years, 4 females, one left handed, 4 males), while 10 participants (22.9 ± 1.5 years, 4 females, 6 males, one left handed) remained in the second group (labeled the Evening-Morning-Evening (EME) group accordingly).

There was no additional reward or remuneration. Participants were required to refrain from daytime naps, alcohol, excessive caffeine-intake, and any other drugs from 24 h before initial training until the end of the experiment. Physical activity (e.g., sport practice) was permitted. All participants were naïve with respect to the criterion task and the research hypotheses.

Duration and quality of each subjects’ sleep during the night of the experiment was assessed with a standardized sleep questionnaire (Goertelmeyer, 1986). There was no indication of poor sleep quality for any of the participants. Also, daytime activities during the wakening retention interval were assessed with a time-line protocol. Again, no peculiarities were observed with respect to any of the subjects.

### Task and Apparatus

**Figure 1:**
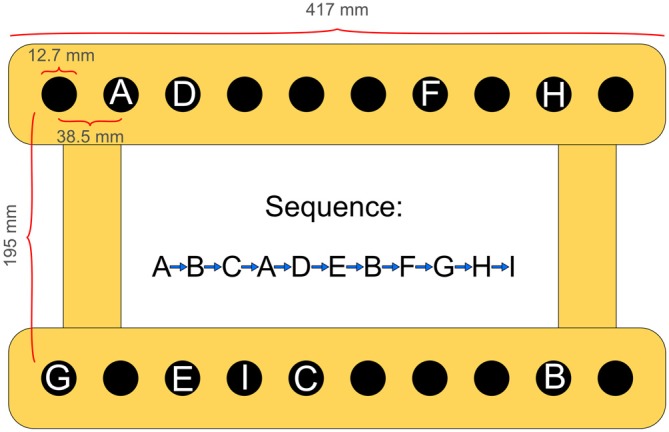
**Experimental apparatus and spatial locations to be reached for one after the other, defining the 10-element arm movement sequence**.

The criterion task required participants to repeatedly carry out a fixed sequence of 10 reaching movements with their non-dominant arm. Subjects were seated comfortably in a height-adjustable chair in front of a table-mounted electronic pegboard and a vertical computer screen with their upper trunk against the backrest. With their hand visible all the time, participants could freely move shoulder, elbow and wrist. On each trial, following a start signal they had to successively fit a small hand-held peg into the respective target-holes (depth: 22.22 mm; diameter: 12.7 mm) on the pegboard (see Figure 1). Thereby they followed a fixed pattern of end-point locations in the horizontal plane, which was void of any apparent regularity. Transport movements differed in amplitude (range: 3.83–33.75 cm) and direction. Precision requirements for all sequence elements amounted to an index of difficulty (ID) of 5.03 (±0.94) on average (Fitts, 1954). According to Fitts, the ID is determined by the equation Log_2_ (2A/W), where A represents the movement amplitude measured from one target center to the other target center and W represents the width of the target area in the direction of the movement.

The sequence to perform was never presented entirely before or during execution. Rather, participants learned the sequence by repeated execution, similarly to a serial reaction time task. During acquisition, targets were visualized one after the other prior to each reaching movement on a computer screen. Correct execution of a sequence element was indicated by a color change of the respective target stimulus from red to green, while the next target symbol was illuminated red. In case of a reaching error, the symbol representing the target that had been missed turned green as well, while the next target was illuminated red. Thus, explicit error control always required participants to compare the peg’s present position on the pegboard to the target position indicated on the screen. As soon as one sequence element was terminated, the next reaching movement had to be started immediately, until the sequence was completed. Once a sequence trial was finished, subjects had to place the peg back into the starting position and prepare themselves for the next trial. After announcing they were ready again participants received an oral start-signal about 1 s later, and then executed the next trial. This procedure was repeated until a block of 10 trials had been accomplished. During recall, no extrinsic information (neither stimulus information nor feedback) was provided. Sequence configuration, raw data assessment and screen display during sequence execution were controlled by means of LMD Software (Wagner: IAT Leipzig, Germany).

### Dependant Measures

Acquisition and recall tests were organized in successive blocks of 10 trials, separated by 30-s resting periods. To prevent any build up of fatigue during acquisition, the resting period following block six was extended to 2.5 min. Performance measures were number of Erroneous Sequences (ES) per trial block (i.e., error rate), and Total Execution Time (TET) per sequence, with TET averaged for each subject across correct sequences in a trial block. TET thereby is inversely proportional to sequence execution speed. Participants were instructed to execute each single sequence-trial as rapidly as possible with as few errors as possible. They were also advised not to speed up performance at the expense of an increasing error rate. Instructions were followed by most of the participants, resulting in marked skewness of the dependent variable ES (i.e., number of ES).

It should be mentioned here that this gross motor task was sufficiently complex and difficult to prevent performance reaching an asymptote within one single practice session. As had been shown previously in a pilot study with eight subjects (23.13 ± 2.1 years, 4 females, 4 males) extensively practicing this same criterion task on three successive days (600 trials altogether; two training sessions of 100 trials per day, stimulus information continuously provided), mean performance (i.e., sequence execution speed, operationalized via TET) continuously increased following a power function, and started to level off only after about 550 trials at about 5.7 s TET on average (unpublished data; Schmitz and Waßmuth, 2013). It also became clear from that study that more than 100 trials would be needed to fully memorize the spatial movement pattern.

### Design and Procedure

**Figure 2:**
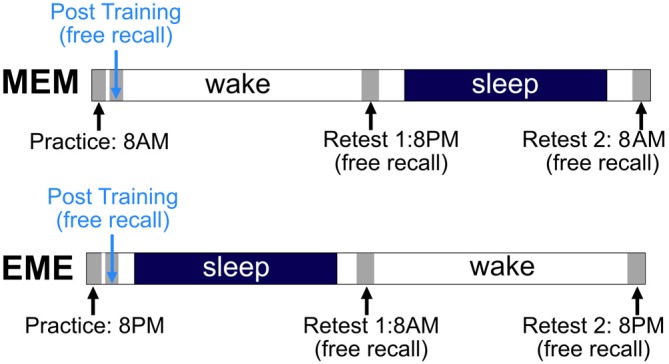
**Experimental paradigm.** For details see text.

After being shortly familiarized with the electronical pegboard and the peg-plugging procedure in general, both experimental groups received initial training of the criterion task (12 blocks of 10 trials each). Both groups then were retested three times in a free-recall condition, namely 15 min after end of practice (Post-Training), and again 12 h (Retest 1) and 24 h later (Retest 2), with each Retest comprising three blocks of 10 trials. The first group to take part in this experiment received initial training in the morning (7–9 a.m.) and was labeled the MEM group accordingly, while the second group practiced in the evening (7–9 p.m.), and was labeled the EME group respectively. Thus, subjects in the MEM-group had a regular night’s sleep during their second 12-h retention interval, those in the EME-group during their first 12-h retention interval (cf. Figure 2). To prevent mental rehearsal of the criterion task during the 15-min retention interval directly following acquisition, participants were asked to read a series of comic stories combining pictures and text. They also were instructed to report on the stories’ content at the end of the respective test session.

### Statistics

Changes in performance during acquisition and retention were analyzed with reference to five different time points, namely “Start of Practice”, “End of Practice”, “Post-Training”, “Retest 1”, and “Retest 2”. Time point-specific performance values were calculated as follows: first, for each subject ES- and TET-measures were averaged across trials per block. Then for each subject and dependent variable, average performance measures were calculated from the first three initial training blocks (Start of Practice, blocks 1, 2 and 3) and from the last three initial training blocks (End of Practice, blocks 10, 11 and 12), while retest measures were calculated from blocks 13, 14, and 15 (Post-Training), 16, 17, and 18 (Retest 1), and 19, 20, and 21 (Retest 2) respectively. Group mean values (medians) were calculated on this basis.

In the presence of small sample sizes and extreme skewness of the dependent variable ES for inferential statistics non-parametric procedures were applied. Accordingly, Friedman test and Wilcoxon test were used for within-group comparisons, while Mann-Whitney *U* test was applied when data were compared across groups. A significance level of *p* < 0.05 was used for all inferential statistics. In case of multiple testing Bonferoni-corrections were applied. As a rule statistical significance was assessed two-tailed, with exact *p*-values being reported. Effect sizes were provided in terms of Cohen’s *r*

$$\left(r\;=\;\frac{\left|z\right|}{\sqrt{N}}\right)\text{ and }\Phi_{c}\left(\Phi_{c}\;=\;\sqrt{\frac{\chi^{2}}{\text{N}(k-1)}}\right)$$

with respect to non-parametric tests (Fritz et al., 2012).

## Results

### Descriptive Data

Performance data (i.e., number of ES and TET) achieved by each group at the respective time points are presented in Table 1.

**Table 1:** **Behavioral data: number of Erroneous Sequences (ES) and Total Execution Time (TET)**.

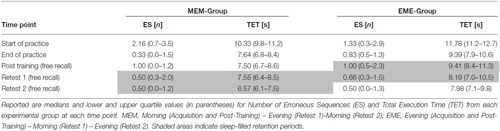

### Acquisition and Transfer to Free Recall

In a first step, changes in performance during acquisition and at transfer to the first free-recall test were determined for both groups. Throughout acquisition, number of *ES* was low on average (*MD* = 0.87) in the MEM-group, but even so from Start of Practice to End of Practice error rate significantly decreased (*Z* = −2.527, *p* = 0.008, Cohen’s *r* = 0.892), as did *TET* (*Z* = −2.521, *p* = 0.008, Cohen’s *r* = 0.890). However, when participants were subjected to the first free-recall test at Post-Training, compared to End of Practice both ES (*Z* = −0.527, *p* = 0.688) and TET (*Z* = −0.280, *p* = 0.844) statistically remained about the same. Also in the EME-group, error rate was low on average throughout acquisition (*MD* = 1.16). While number of *ES* this time did not change significantly from Start of Practice to End of Practice (*Z* = −1.602, *p* = 0.129), *TET* again significantly decreased (*Z* = −2.521, *p* = 0.008, Cohen’s *r* = 0.890). When participants underwent the first free-recall test at Post-Training, compared to End of Practice both ES (*Z* = −1.266, *p* = 0.258) and TET (EME: *Z* = −0.968, *p* = 0.375) statistically remained about the same again.

Thus, both groups during acquisition significantly improved sequence execution speed and also somewhat reduced error rate, while transfer from an informational guided practice condition to free recall 15 min later did not yield any performance decrements. On the whole, error rate was real low throughout the whole experiment in either group, and there was no speed-accuracy trade-off across time points.

### Retention (Free Recall Only)

In a second step possible performance changes during retention under free-recall conditions had to be determined. According to our theoretical considerations it was of specific interest, if possible performance changes during the sleep-filled retention intervals were any different from performance changes during the respective wake intervals. Considering the small sample sizes, and in order to achieve sufficient statistical power, we applied the following procedure: data of both experimental groups were combined and subjected to the respective statistical tests conjointly, so that pre- and post-wake performance data of all 18 participants could be compared directly, and pre- and post-sleep performance data of all 18 participants could be compared directly, too. Due to the circadian offset of 12 h between both experimental groups the combined pre- and post-wake interval and pre- and post-sleep interval data for each dependent variable had to be compared in two separate test runs. It has been argued that these two tests were conceptually related. Therefore the level of significance in these cases was and set at *p* = 0.025 (two-tailed) following Bonferoni correction.

**Figure 3:**
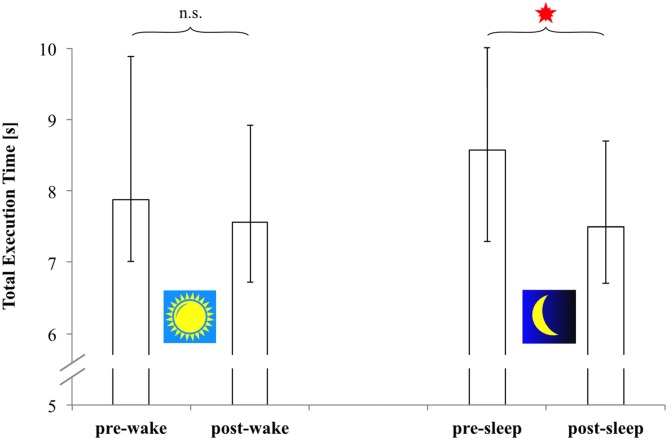
**Total execution time (seconds; correct sequences only) of all 18 subjects (Morning-Evening-Morning-group and Evening-Morning-Evening-group combined) at free recall.** Presented are measures based on the combined data from both groups’ pre- and post-wake retention tests (left panel), and from both groups’ pre- and post-sleep retention tests (right panel). Open bars: medians; Error bars: upper and lower quartiles. *Significant difference of group medians (*p* = 0.001).

The following results now refer to the combined data of both groups. According to the respective Wilcoxon tests, *error rate* (ES) remained the same across both retention intervals (wake retention interval: *p* = 0.404; sleep-filled retention interval: *p* = 0.106). However, *sequence execution time* (TET) *significantly decreased* during the *sleep-filled* retention interval (*Z* = −3.245, *p* = 0.001, Cohen’s *r* = 0.540), but not so during the wake retention interval (*Z* = −1.894, *p* = 0.060, Cohen’s *r* = 0.315). The respective TET-data are depicted in Figure 3.

Thus, regarding our total sample (*N* = 18) the following became evident: *TET* *significantly decreased* (i.e., sequence execution speed increased) during the *sleep-filled* 12-h retention interval, but remained statistically unchanged during the respective 12-h wake interval. *Error rate (ES)*, on the other hand, remained completely unaffected by the respective treatment conditions throughout retention. So also during retention there was no indication of any speed-accuracy trade-off. These findings were well in line with our theoretical expectations of sleep-dependent offline-gains in sequence execution speed. They were also corroborated by statistical analysis at the single group level (see “Supplementary Material”).

## Discussion

The present study was intended to test the notion of sleep-related offline learning coming into effect in a sequentially structured gross motor task after only limited practice and under free recall requirements. These are conditions common to many applied areas in the motor learning domain. From a theoretical point of view, any offline improvements in performance observed at retention under these conditions can be attributed solely to an enhanced sequence memory, since continued online learning at retests is effectively prevented by the absence of criterion-related stimulus information. In traditional motor learning experiments, only terminal feedback is usually removed to prevent further learning. But as long as stimulus information is still present at retesting (like e.g., in the typical serial reaction time task), continued updating of sequence memory on grounds of externally provided information cannot be prevented. From an ecological point of view such testing conditions are not likely to reliably engage retrieval strategies relevant to many real-life situations in the field.

In the present study a 10-element sequence of reaching movements was used for a criterion task. Participants executed this sequence on an electronic pegboard with their unrestrained non-dominant arm, thereby following a fixed spatial pattern in the horizontal plain. The pattern had no apparent regularities. The sequence had to be carried out as rapidly and with as few errors as possible. Dependent variables were number of ES, and total sequence execution time. These performance measures thus represented error rate and sequence execution speed. Two groups of altogether 24 subjects initially learned this sequence for a total of 120 trials either in the morning (MEM-group) or in the evening (EME-group). Performance was retested 15 min post training, as well as 12 h and 24 h later. Thus, each group was provided with one sleep-filled and one wake retention interval. All three retests required free recall of the criterion sequence.

At the end of practice all subjects had more or less explicit knowledge of the sequence they had learned, and were using different retrieval strategies at (re)testing. This can be concluded from subjects’ verbal reports given at the end of the experiment. However, to which extent participants used cognitive retrieval strategies or more procedural aspects of the motor task in question (cf. Hikosaka et al., 1999) cannot be decided. At any case, six subjects (four in the MEM- and two in the EME-group) were unable to reproduce the initially learned sequence under free recall conditions, even when they tried to explicitly remember the sequence. These subjects were excluded from further analysis.

In the remaining 18 subjects error rate was low right from the beginning and dropped to well below one erroneous sequence per block of 10 trials at the end of practice. Sequence execution speed improved significantly in both groups during acquisition. During retention error rate did not change any more (no group differences). Total sequence execution time during retention significantly *decreased* following sleep, but not following wake. This held true for the total sample, and could also be corroborated for each group separately (cf. “Supplementary Material”). Throughout the experiment there was no speed-accuracy trade-off.

It should be noted that sequence execution time at the end of practice in both groups was still well above (at least 2 s) asymptotic performance level. The performance asymptote for this same task has been determined in a previous study after three days of continued practice by eight young subjects of comparable age (Schmitz and Waßmuth, 2013). Therefore it seems unlikely that global differences in sequence execution speed between experimental groups could have biased the sleep-related improvements in performance found at retention to any relevant extent. Also, this finding of sleep-related motor performance improvement was independent from retention interval duration and time of day of learning: the EME-group initially acquired the criterion sequence in the evening and was afforded sleep during the first 12 h retention period. The MEM-group to the contrary learned the sequence in the morning and slept during the second 12 h retention period. All in all these results corroborate recent findings of sleep-related motor offline learning in a very similar task, however with the same stimulus information provided at retention as well as during the initial learning phase (Malangré et al., 2014).

It should be mentioned that in the EME-group, following significant sleep-dependent offline improvement, sequence execution time also decreased somewhat during the second (the wake) retention interval. This effect is close to significance (*p* = 0.064, Cohen’s *r* = 0.597; see “Supplementary Material”), and was not observed in the MEM-group. From this one might conjecture that sleep-dependent consolidation mechanisms are still in process during the following wakening period, while this is not the case during the wakening period prior to sleep. This aspect certainly requires closer consideration in the future.

In this context, also the following observation might be of particular interest: in a pilot study (unpublished data) we conducted in our laboratory preceding the experiment presented in this article, two randomized groups of participants (all students at the department of sport science) practiced the same criterion task as was used in our present study either in the morning (ME-group; 21.0 ± 2.4 years; 5 females; 4 males) or in the evening (EM-group; 21.0 ± 0.98 years; 4 females; 7 males) for 120 trials, and were retested under free recall conditions 12 h later, i.e., on the same evening or on the next morning respectively. Note that there was no early free recall test shortly following acquisition. During acquisition total sequence execution time significantly decreased in either group from 9.82 s on average to 7.53 s on average. But then in this pilot study at free recall seven out of the nine subjects in the ME-group were unable to reproduce the criterion sequence after their 12 h wakening interval. Obviously during a 12 h wake retention interval they had forgotten essential sequence components (i.e., certain elements and/or order of elements). To the contrary only two out of the eleven subjects in the EM-group failed to recall the sequence after their 12-h sleep-filled retention interval. Thus, sleep appeared to prevent sequence memory to deteriorate. Also, and different from our present results, in the absence of an early free-recall test in the remaining nine subjects of the pilot-study’s EM-group sequence execution speed at free recall following a night of sleep appeared to be stabilized, but not improved as compared to performance at the end of acquisition. Thus, it could be argued that withdrawing stimulus information and feedback opportunity during testing might have hidden possible sleep-dependent performance improvements.

Thus, implementation of an early free recall test (Post-Training) in our present experiment not only provided for an appropriate datum point subjects’ performance at the two later free recall tests could be related to i.e., transfer-appropriate processing; cf. Lee (1988). We conjecture that it also served as a means to effectively reduce the tendency for sequence representation to decay over a 12 h wakening period, and to provide a basis for subsequent enhancement of sequence memory during sleep. We assume that the necessity of free recall soon after acquisition stabilizes and even considerably elaborates the multifaceted sequence representation still intact at that point of time. This positive effect of early retesting on long term retention has recently been found for verbal material (Roedinger and Karpicke, 2006) as well as for effector transfer in motor sequence learning, which is indicative for the generalization of the abstract spatial sequence pattern (Boutin et al., 2013). Thus, testing conditions not only boosts memory when learners are allowed to practice between testing sessions as in the study of Boutin et al. (2013), but early testing under free recall conditions might also shape sequence memory so to enhance later retention.

All in all, while with the present experiment we successfully corroborated and extended recent findings on sleep-related offline learning in gross motor sequence learning tasks, there are also clear limitations to our study in that sample size was rather small, and subjects were not randomly assigned to the experimental groups.

## Author Contributions

AM and KB contributed extensively and equally to the work presented in this manuscript, developed the research topic and designed the experiment, prepared, analyzed data and discussed results, wrote the article and discussed and commented on the manuscript at all stages. AM supervised data collection.

## Conflict of Interest Statement

The authors declare that the research was conducted in the absence of any commercial or financial relationships that could be construed as a potential conflict of interest.
