# Supplementary material for: Sleep-Related Offline Improvements in Gross Motor Task Performance Occur Under Free Recall Requirements
Source: Front Hum Neurosci. 2016 Mar 29;10:134. doi: 10.3389/fnhum.2016.00134 (PMC4809884; doi:10.3389/fnhum.2016.00134)
Supplement: Supplementary file 1 [file Presentation_1.pdf]

Sleep-related offline improvements in gross motor task performance occur under free recall requirements - Supplemental material

Andreas Malangré and Klaus Blischke

Results at the single group level

Here, for each group descriptive data on the development of error rate (ES) and sequence execution time (TET) across acquisition (trial blocks 1 through12) and retention (trial blocks 13 through 21) are shown. Also, detailed inferential statistics on both dependent measures regarding the retention period under free recall conditions are presented at the single group level, complementing the more global approach followed in the main article.

1.1 MEM condition (N = 8)

Performance across trial blocks, averaged across all subjects in the MEM-condition who successfully completed the whole experiment are presented in Figure 4, covering both acquisition and retention.

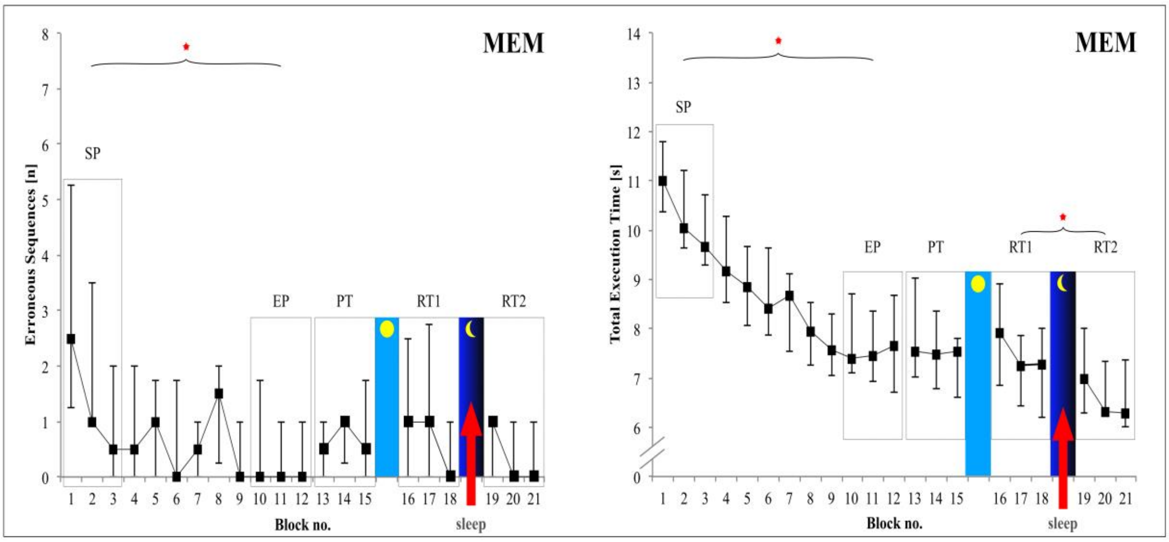

Figure 4: MEM-group. (a) Left panel: Number of Erroneous Sequences (ES) per trial block: acquisition (blocks 1 through 12) and subsequent retests (blocks 13 through 21). Symbols represent group medians per trial block; error bars: upper and lower quartiles. (b) Right panel: Total Execution Time (TET; seconds) per trial block (correct sequences only): acquisition (blocks 1 through 12) and subsequent retests (blocks 13 through 21). Symbols represent group medians per trial block; error bars: upper and lower quartiles. Time Points (left and right panel): Start of Practice; End of Practice; Post-Training (early free-recall test); Retention Test 1; Retention Test 2.

According to a Friedman test calculated across the three relevant time points "Post-Training", "Retest 1", and "Retest 2", *error rate (ES)* did not change significantly in the MEM-group throughout the total 24-hrs retention period (χ^2 = 2.263, p = .345). This was confirmed by Wilcoxon tests calculated separately for each 12-hr retention interval (Post-Training – Retest 1: Z = -.426, p = .750; Retest 1 – Retest 2: $Z_{]}$ = -1.023, p = .375). *Total sequence execution time (TET)*, however, in this group *significantly decreased* across all three time points according to the respective Friedman test (χ^2 = 13.000, p < .001, Φ_c = .901). In order to decide whether the observed offline-gains in sequence execution speed were indeed sleep- or just time-dependent, this result was closer analyzed by means of Wilcoxon tests

calculated separately for each 12-hr retention interval. It appeared that *TET decreased significantly* only during the MEM-group's *second* (i.e. the *sleep-filled*) retention interval, but not during the first (i.e. the wakening) 12-hrs retention interval (Post-Training – Retest 1: Z = -.560, p = .641, Cohen's r = .198; Retest 1 – Retest 2: Z = -2.521, p = .008, Cohen's r = .890).

1.2 EME condition (N = 10)

Performance across trial blocks, averaged across all subjects in the EME-condition who successfully completed the whole experiment are presented in Figure 5, covering both acquisition and retention.

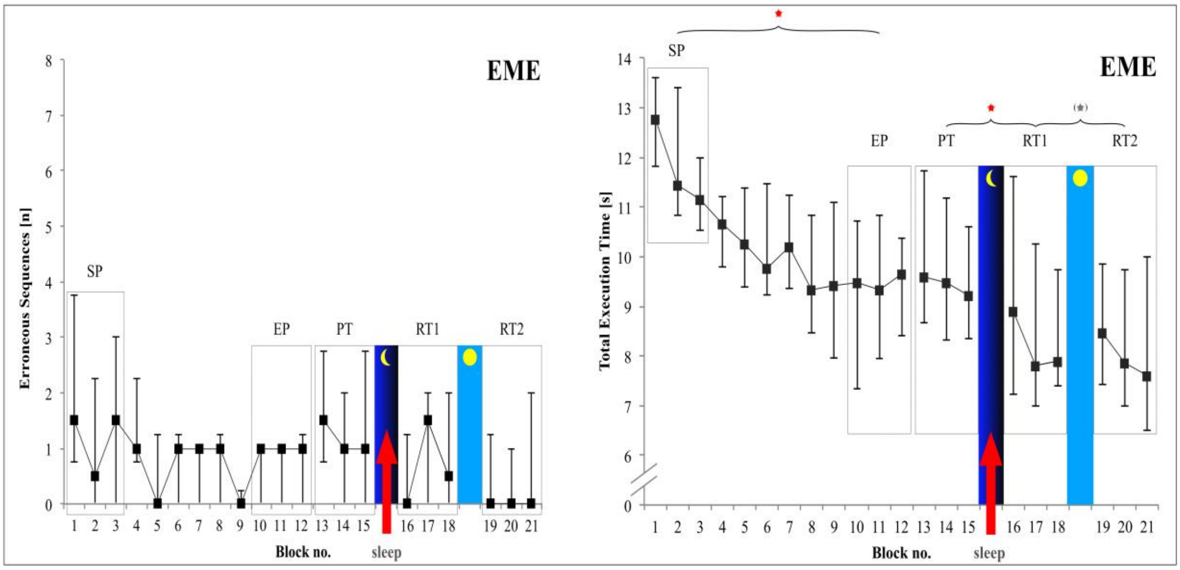

Figure 5: EME-group. (a) Left panel: Number of Erroneous Sequences (ES) per trial block: acquisition (blocks 1 through 12) and subsequent retests (blocks 13 through 21). Symbols represent group medians per trial block; error bars: upper and lower quartiles. (b) Right panel: Total Execution Time (TET; seconds) per trial block (correct sequences only): acquisition (blocks 1 through 12) and subsequent retests (blocks 13 through 21). Symbols represent group medians per trial block; error bars: upper and lower quartiles. Time Points (left and right panel): Start of Practice; End of Practice; Post-Training (early free-recall test); Retention Test 1; Retention Test 2.

As was shown by a Friedman test calculated across the three relevant time points "Post-Training", "Retest 1", and "Retest 2", also in the EME-group number of *Erroneous Sequences (ES)* did not change significantly across the total 24-hrs retention period (χ^2 = 2.889, p = .244). This was confirmed again by Wilcoxon tests calculated separately for each 12-hr retention interval (Post-Training – Retest 1: Z = -1.550, p = .141; Retest 1 – Retest 2: Z = -1.388, p = .176). *Total execution time (TET)*, however, according to the respective Friedman test *significantly decreased* throughout the total 24-hrs retention period in the EME-group as well (χ^2 = 12.800, p = .001, Φ_c = .80). Again Wilcoxon tests were calculated separately for each 12-hr retention interval in order to decide whether these offline-gains in execution speed were sleep- or just time-dependent. As it turned out, *TET significantly decreased* during the EME-group's *first* (i.e. the *sleep-filled*) 12-hrs retention interval, but not during the second (i.e. the wakening interval) any more (Post-Training – Retest 1: Z = -2.395, p = .014, Cohen's r = .758; Retest 1 – Retest 2: Z = -1.886, p = .064; Cohen's r = .597).
